# Potentiometric Surfactant Sensor Based on 1,3-Dihexadecyl-1*H*-benzo[*d*]imidazol-3-ium for Anionic Surfactants in Detergents and Household Care Products

**DOI:** 10.3390/molecules26123627

**Published:** 2021-06-14

**Authors:** Nikola Sakač, Dubravka Madunić-Čačić, Dean Marković, Lucija Hok, Robert Vianello, Bojan Šarkanj, Bojan Đurin, Krunoslav Hajdek, Božo Smoljan, Stjepan Milardović, Brunislav Matasović, Marija Jozanović

**Affiliations:** 1 Faculty of Geotechnical Engineering, University of Zagreb, 42000 Varaždin, Croatia; dubravka.madunic-cacic@saponia.hr; 2 Saponia Chemical, Pharmaceutical and Foodstuff Industry, Inc., 31000 Osijek, Croatia; 3 Department of Biotechnology, University of Rijeka, 51000 Rijeka, Croatia; dean.markovic@biotech.uniri.hr; 4 Division of Organic Chemistry and Biochemistry, Ruđer Bošković Institute, 10000 Zagreb, Croatia; lucija.hok@irb.hr (L.H.); robert.vianello@irb.hr (R.V.); 5 Department of Food Technology, University North, 48000 Koprivnica, Croatia; bsarkanj@unin.hr; 6 Department of Civil Engineering, University North, 42000 Varaždin, Croatia; bdjurin@unin.hr; 7 Department of Packaging, Recycling and Environmental Protection, University North, 48000 Koprivnica, Croatia; krunoslav.hajdek@unin.hr (K.H.); bozo.smoljan@unin.hr (B.S.); 8 Faculty of Chemical Engineering and Technology, University of Zagreb, 10000 Zagreb, Croatia; stjepan.milardovic@fkit.hr; 9 Department of Chemistry, University of Osijek, 31000 Osijek, Croatia; brunislav.matasovic@kemija.unios.hr

**Keywords:** anionic surfactants, sensor, potentiometry, detergents, nonionic surfactants, household detergents, molecular dynamics simulations

## Abstract

A 1,3-dihexadecyl-1*H*-benzo[*d*]imidazol-3-ium-tetraphenylborate (DHBI-TPB) ion-pair implemented in DHBI-TPB surfactant sensor was used for the potentiometric quantification of anionic surfactants in detergents and commercial household care products. The DHBI-TPB ion-pair was characterized by FTIR spectroscopy and computational analysis which revealed a crucial contribution of the C–H∙∙∙π contacts for the optimal complex formation. The DHBI-TPB sensor potentiometric response showed excellent analytical properties and Nernstian slope for SDS (60.1 mV/decade) with LOD 3.2 × 10^−7^ M; and DBS (58.4 mV/decade) with LOD 6.1 × 10^−7^ M was obtained. The sensor possesses exceptional resistance to different organic and inorganic interferences in broad pH (2–10) range. DMIC used as a titrant demonstrated superior analytical performances for potentiometric titrations of SDS, compared to other tested cationic surfactants (DMIC > CTAB > CPC > Hyamine 1622). The combination of DHBI-TPB sensor and DMIC was successfully employed to perform titrations of the highly soluble alkane sulfonate homologues. Nonionic surfactants (increased concentration and number of EO groups) had a negative impact on anionic surfactant titration curves and a signal change. The DHBI-TPB sensor was effectively employed for the determination of technical grade anionic surfactants presenting the recoveries from 99.5 to 101.3%. The sensor was applied on twelve powered samples as well as liquid-gel and handwashing home care detergents containing anionic surfactants. The obtained results showed good agreement compared to the outcomes measured by ISE surfactant sensor and a two-phase titration method. The developed DHBI-TPB surfactant sensor could be used for quality control in industry and has great potential in environmental monitoring.

## 1. Introduction

Tensides or surfactants are surface active agents. There are four main types of surfactants: anionic, cationic, nonionic and amphoteric. They are used for washing, cleaning and disinfection in broad varieties of industries [1]. Rapid industrialization, population growth and increased standard of living have caused constant growth of surfactant demands. In 2019, the global surfactant market was estimated to be USD 39,901 million with a predicted CAGR of 4.5% between 2020 and 2025 [2]. Anionic surfactants form the biggest part of the global production (about 70%) with a forecasted CAGR of 4.2% for the period from 2020 to 2027 [2]. The growing demand for anionic surfactants is the result of the increased need for home care, personal care applications and recently, for electric vehicles [2]. Nonionic surfactants are amphipathic molecules. They consist of a lipophilic part (long alkyl chain or fatty acid) and a hydrophilic part (ethylene oxide) of varying length. Nonionic surfactants do not ionize in water.

In household applications, anionic surfactants are used in detergents or emulsifiers for a variety of home products for washing and cleaning. They are utilized regularly in combination with nonionic surfactants to enhance cleaning properties. They are also employed as wetting agents in oil-in-water systems in beauty products to achieve stable emulsions with the pigments and fillers dissolved in the water phase [3].

Because of the reduction of the water tension, prevention of the gas exchange on the water surface and eutrophication, anionic surfactants have a negative influence on the environment. They also induce the disintegration of cell membranes. Thus, the development of new, simple, reliable and robust methods to control the levels of anionic surfactants in a variety of products during their production and quality control as well as in the environment is paramount and essential.

The classical method for the determination of low concentrations of anionic surfactants is based on methylene blue active substances (MBAS) [4], whereas high concentrations can be obtained by two-phase titration [5]. Both methods are widely used but they have many drawbacks including their time-consuming character employment of the toxic chemicals and requirement of a well-trained and experienced analyst. In contrast, ion selective surfactant sensors could overcome the mentioned drawbacks, as they are simple, low-cost, reliable and fast.

Except for the solid state surfactant sensors [6,7,8], they are usually based on the PVC liquid-type sensor membrane incorporated in the electrode body or coated on a wire [9]. Sensor membrane usually consists of a high molecular weight PVC and a plasticizer in a weight ratio 1:2 and an ionophore. The plasticizer softens the rigid PVC and allows the ionophore to be dissolved in a high lipophilic medium. The ionophore is an ion-pair synthesized by the reaction of high molecular weight anionic and cationic ions and/or surfactants [10,11]. In order to prevent leaching in water and to decrease the lifetime, the ionophore should have very low solubility in water and high lipophilicity, whereas to increase the sensitivity and the lifetime, high stability is needed [12], leading to the positive influence on the sensor properties such as higher sensitivity and broader effective concentration range.

The development of the new ionophores is of crucial importance for the advancement of novel surfactant sensors with better properties. Recently, our group [12,13] and others [14] showed that the increase of the active surface and electrotransfer may be achieved by the employment of the nanomaterials as ionophores that also provide higher signal stability and lifetime. As nonionic surfactants have a negative influence on potentiometric titrations and the end-point detection [15], it is important to investigate the influence of nonionic surfactants on potentiometric curve shape, size and especially, the detection of the end-point.

Recently we synthesized 1,3-dihexadecyl-1*H*-benzo[*d*]imidazol-3-ium-tetraphenylborate (DHBI-TPB) and used it as a new long-chain quaternary ammonium cation with high amphipathic character to detect cationic surfactants in personal care products and disinfectants [16]. The aim of this communication is to investigate the performances of developed DHBI-TPB surfactant sensor for the detection and quantification of the anionic surfactants. The studies of the influence of nonionic surfactants on the device performances were also performed. Finally, newly developed DHBI-TPB electrode was employed for the quantification of anionic surfactants in detergents and household care products.

## 2. Results

### 2.1. Computational Analysis

Computational analysis was performed to provide an insight into the dynamics and behavior of the DHBI^+^ cation in the aqueous solution, and to identify intermolecular interactions responsible for its complex formation with TPB^−^, particularly focused on characterizing the DHBI-TPB complex through electronic, geometric and energetic features.

Despite having a rigid benzimidazole skeleton, DHBI^+^ is a very flexible molecule, attributed to its two unconstrained lipophilic C-16 chains, whose flexibility over the anionic TPB^−^ counterpart is clearly evident in the calculated RMSD display (Figure S2). As the following discussion will show, it is precisely this feature of DHBI^+^ that we identified as crucial for its efficient recognition of TPB^−^.

**Figure 1:**
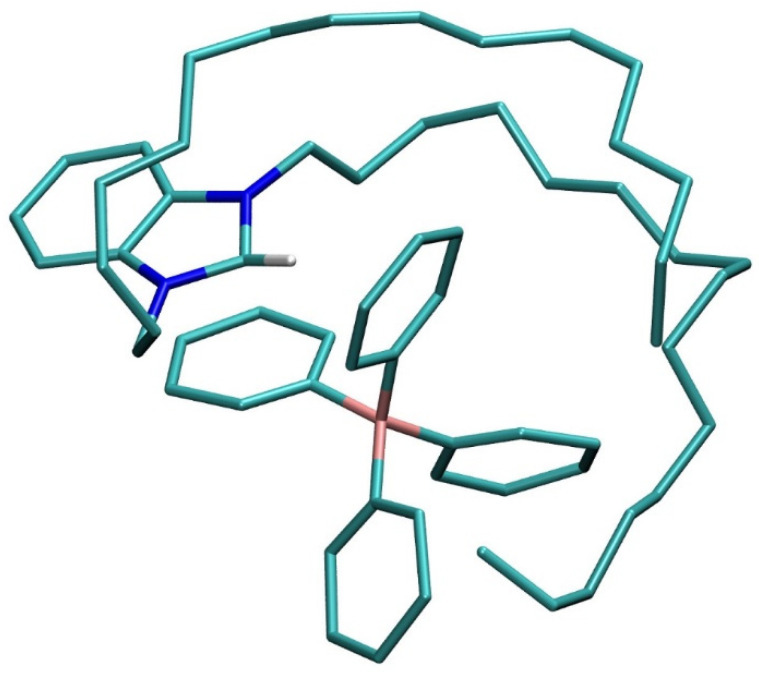
The representative structure of the DHBI-TPB complex in the aqueous solution, with hydrogen atoms omitted due to clarity.

DHBI-TPB complexation is a favorable event, yet the adduct formation and its dissociation into components exchange over the simulation time. As an illustration, the relevant B(TPB)∙∙∙N(DHBI) distances stretch well beyond 40 Å, while assuming values below 7 Å in around 22% of the obtained structures (Figure S3). Still, the MM-PBSA binding free energy between DHBI^+^ and TPB^−^ is exergonic at Δ*G*_BIND_ = −5.0 kcal mol^−1^, thus confirming the feasibility of the process. To put this number in a proper context and given the highly favorable analytical features of the DHBI-TPB sensor, the calculated value appears optimal as it needs to assure the stability of the ion-pair, but also the reversibility of the salt formation and the potential exchange with other anionic analytes for sensing purposes. The representative structure of the complex (Figure 1) reveals two types of favorable interactions, namely (i) the π–π stacking contacts between benzimidazole in DHBI^+^ and one of the phenyl rings in TPB^−^, and (ii) a range of C–H∙∙∙π interactions that both C-16 chains are forming with other phenyl rings in TPB^−^. Moreover, the corresponding B(TPB^−^)∙∙∙N(DHBI^+^) distances assume 4.8 and 5.1 Å, being in good agreement with the relevant overall RDF graph that predicts the largest number of these interactions occurring at 5.4 Å (Figure S4), thus confirming the validity of the presented structure as representative.

Given the opposing charges on both constituents, one could assume that electrostatic charge–charge attraction is governing the binding. Yet, this does not appear to be the case following from the atomic charge analysis (Figure S5). It turns out that, prior to binding, the benzimidazole unit within DHBI^+^ accumulates only a third of the excess positive charge (0.33 |e|), while the rest is deposited within the C-16 chains—a situation which is not changed even in the formed complex. In fact, upon the DHBI-TPB adduct formation, only 2% of the charge is transferred among components, as the total atomic charges on DHBI^+^ and TPB^−^ are +1.02 and −0.98 |e|, which clearly excludes electrostatic charge–charge attraction as predominant for the complex stability. Along these lines, by analyzing structures during simulations and extracting those where the centers of mass among benzimidazole in DHBI^+^ and either of the phenyls in TPB^−^ are found below 4 Å, it was revealed that only in 6.3% of structures did there occur notable π–π stacking contacts, which renders this type of interaction as moderately important as well.

In contrast, what appears to be crucial for the DHBI-TPB complex formation are C–H∙∙∙π interactions between both C-16 chains in DHBI^+^ and phenyls in TPB^−^ (Figure 1). To confirm that, we have repeated MD simulations with two modified DHBI^+^ systems, in which, firstly, one C-16 chain is replaced by the methyl group, and, secondly, both C-16 chains are substituted by methyls. In those cases, the matching binding free energy was reduced by 0.7 kcal mol^−1^ to Δ*G*_BIND_ = −4.3 kcal mol^−1^, for the system with one C-16 chain, and further, by as much as 3.3 kcal mol^−1^ down to Δ*G*_BIND_ = −1.0 kcal mol^−1^, for the system without C-16 chains. The latter strongly underlines the crucial importance of the lipophilic chains for the design of useful analytical devices through allowing the formation of efficient ion-pair sensors.

### 2.2. Sensor Characterization

#### 2.2.1. Sensor Response to Anionic Surfactants

A Nernst Equation (1) describes the sensor potential response (E) on anionic surfactants concentration ($a_{A^{-}}$):

(1) $$E=E^{o}-S\times loga_{A^{-}}$$

where

E= electromotive force of the system
$E^{o}$= constant potential term
S= sensor slope
$a_{A^{-}}$= anionic surfactant sensor activity

Response characteristics of a DHBI–TPB surfactant sensor were observed against the SDS and DBS anionic surfactants in deionized water and 0.01 M Na_2_SO_4_ solution. The Na_2_SO_4_ solutions were used to observe the response in high ionic strength solution. Detailed data on the response characteristics and statistics are presented in Table 1. Notably, the high ionic strength did not lead to the strong effect on the surfactant sensor characteristics, as the slope values, in mV per decade of activity, are near-Nernstian for SDS (60.1 ± 0.5 and 59.7 ± 0.4 mV/decade) and DBS (58.4 ± 0.6 and 58.7 ± 0.5 mV/decade), both in water and Na_2_SO_4_ solution. The correlation coefficients (R^2^) were in the range from 0.9993 to 0.9995, meaning the signal was stable, with broad linear trend. Useful linear concentration range for SDS in water was 4.6 × 10^−7^ to 5.1 × 10^−3^ M (LOD 3.2 × 10^−7^ M) and 6.8 × 10^−7^ to 5.1 × 10^−3^ (LOD 4.2 × 10^−7^ M) in Na_2_SO_4_ solution, respectively. Useful linear concentration ranges for DBS in water and Na_2_SO_4_ were 8.9 × 10^−7^ to 4.1 × 10^−3^ M, with LOD 6.1 × 10^−7^ M.

#### 2.2.2. pH Influence and Interference Study

The pH influence on the response characteristics of the DHBI-TPB surfactant sensor was measured in a broad pH range from 2 to 12. The signal change was stable and was not affected by the pH change. Small changes in the signal were observed for pH 10 and higher. The latter observations indicate that the DHBI-TPB sensor could also be used in determination of anionic surfactants in commercial formulations.

Interference study was performed by incremental addition of SDS in 0.01 M interfering ion solution. A fixed interference method [17] to calculate the logarithm of the selectivity coefficient ($log\;K_{An_{i.}^{-}}^{pot}$) for all selected interfering anions commonly employed in the commercial products formulations was used (Table 2). As presented, the concentration of SDS was in the range from 4 × 10^−6^ to 4 × 10^−3^ M. The DHBI-TPB surfactant sensor showed great selectivity for anionic surfactant SDS for all investigated inorganic and organic anions.

### 2.3. Potentiometric Titrations

#### 2.3.1. Titrant Selection

**Figure 2:**
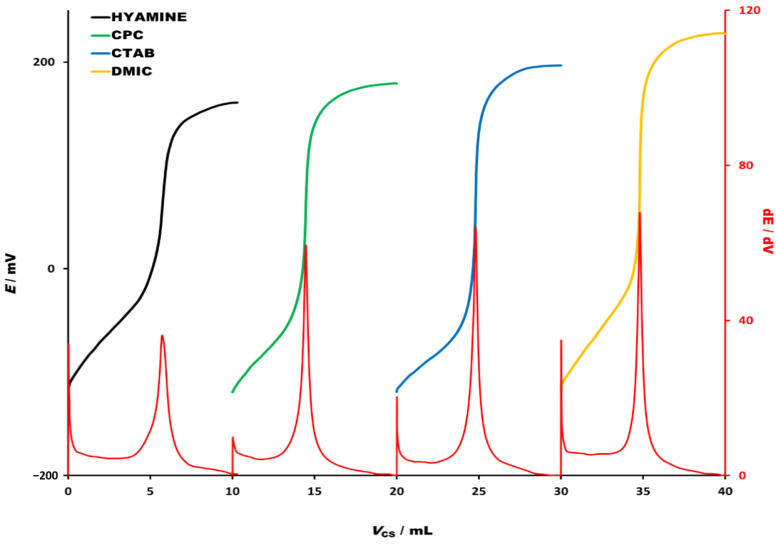
Potentiometric titration curves for titration of SDS (4 × 10^−3^ M) with four different cationic surfactants (4 × 10^−3^ M) used as a titrant: Hyamine 1622 (black line), CPC (green line), CTAB (blue line) and DMIC (yellow line). Corresponding first derivatives are presented in red lines below titration curves. The titration curves and their first derivatives are rearranged for the sake of clarity.

In order to select the best performing titrant, four analytical grade cationic surfactants—Hyamine 1622, CPC, CTAB and DMIC (all 4 × 10^−3^ M), were used for the titrations of SDS (4 × 10^−3^ M). The titration curves and their first derivatives (ΔE/ΔV) are presented in Figure 2. First derivatives for all titration curves showed high potential change in equivalence point. Although DMIC had superior properties as the highest signal change (potential jump in equivalence point) and the highest first derivative signal change was observed, the titration curves for Hyamine 1622, CPC and CTAB showed useful analytical performances as well. In general, falling properties of cationic titrants were DMIC > CTAB > CPC > Hyamine 1622. Since DMIC was proven to be the superior titrant, not only for high anionic surfactant concentrations, but also for very low concentrations, the latter was used for further studies. The studied concentrations of anionic surfactants were in the range usually found in commercial product formulations.

#### 2.3.2. Titrations of Alkane Sulfonate Homologues

**Figure 3:**
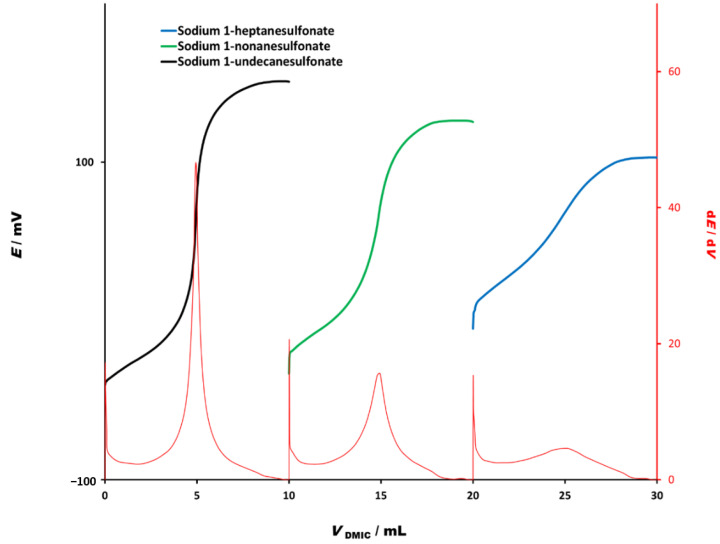
Potentiometric titration curves for titration of analytical grade alkane sulfonate homologues (4 × 10^−3^ M) with heptanesulfonate (**7**), nonansulfonate (**9**) and undecanesulfonate (**11**) with DMIC (4 × 10^−4^ M).

Alkane sulfonate homologues with short chain-lengths are exceptionally soluble in water and therefore, are often difficult to analyze by potentiometric titrations as well as by two-phase titrations. DMIC (4 × 10^−4^ M) showed excellent analytical properties (Figure 2) and was tested as titrant in potentiometric titration of alkane sulfonate homologues with chain-lengths from 7 to 11 having concentration 4 × 10^−4^ M. As shown in Figure 3, the titration curves had defined and sharp inflexion points with high potential signal change and well-defined first derivative signal change, except for the shortest chain heptanesulfonate. However, the titration curve for heptanesulfonate still possesses a visible inflexion point and useful potential signal change, with moderately defined first derivative signal change. 

#### 2.3.3. The Influence of Nonionic Surfactant on Titrations

Commercial formulations often contain a mixture of anionic surfactants and nonionic surfactants, such as ethoxylated nonionic surfactants (EONSs). Nonionic surfactants are usually used to enhance washing properties of commercial products. Thus, we also examined the influence of EONSs on the potentiometric titrations of anionic surfactants. We considered the different molar ratio of SDS and EONS having 10 EO groups as well as S; the influence of the EONS’ number of EO groups on the potentiometric titrations was studied.

**Figure 4:**
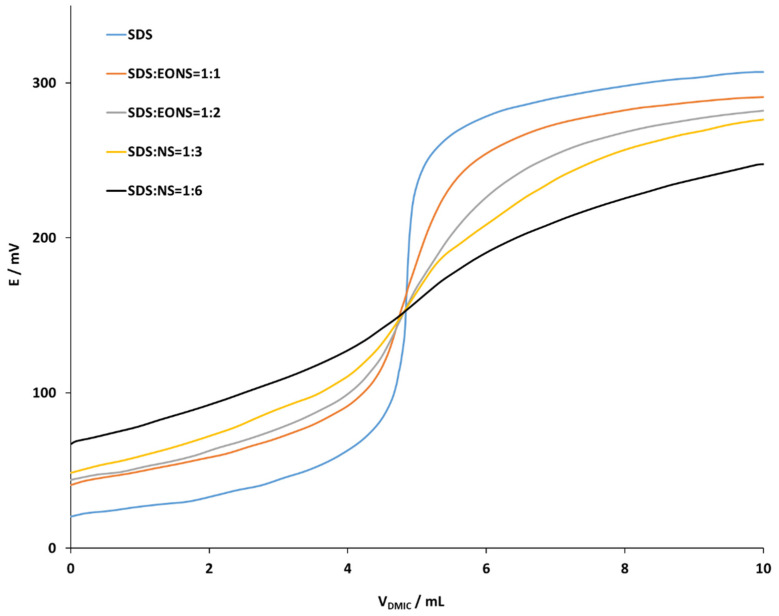
Potentiometric titration curves for titration of mixtures with different molar ratio of SDS (4 × 10^−3^ M) and 10 EO EONS using DMIC (4 × 10^−3^ M) as a titrant and a DHBI–TPB surfactant sensor as an end-point indicator. The titration curves are rearranged for the sake of clarity in the following order: SDS:EONS ratio 1:0 (blue line), 1:1 (red line), 1:2 (grey line), 1:3 (yellow line) and 1:6 (black line).

**Figure 5:**
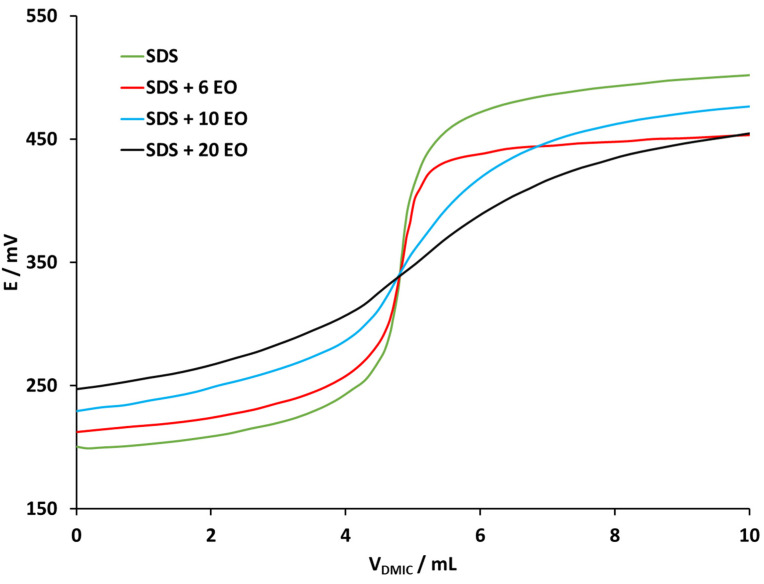
Potentiometric titration curves for titration of the mixture of SDS (4 × 10^−3^ M) and EONS with a different number of EO groups at fixed DS: EONS molar ratio (1:2) with the DMIC (4 × 10^−3^ M) as a titrant and a DHBI–TPB surfactant sensor as an end-point indicator. The titration curves are rearranged for the sake of clarity in the following order: only SDS (green line), SDS with 6 EO groups (red line), SDS with 10 EO groups (blue line), SDS with 20 EO groups (black line).

Selected EONS having 10 EO groups employing cationic surfactant DMIC (4 × 10^−3^ M) as a titrant and a DHBI–TPB surfactant sensor as an end-point indicator are presented in Figure 4. Potentiometric titration curves for titration of mixtures with different molar ratio of anionic surfactant SDS (4 × 10^−3^ M) are presented in Figure 5. Selected SDS to EONS molar ratios were 1:0, 1:1, 1:2, 1:3 and 1:6. Increased molar ratio of EONS had a significant negative influence on the shape, inflexion and the size of potentiometric titration curves. With increased EONS molar ratio, the titration curve appeared more flattened. This might negatively influence the direct potentiometric determination of the anionic surfactants in the samples.

Potentiometric titration curves for titration of the mixtures of anionic SDS (4 × 10^−3^ M) and nonionic surfactant EONS with different number of EO groups (0, 6, 10 and 20 EO) at fixed DS to EONS molar ratio (1:2), with cationic surfactant DMIC (4 × 10^−3^ M) as a titrant and a DHBI–TPB surfactant sensor as an end-point indicator, are presented in Figure 5. Increased number of EO groups had a significant negative influence on the shape, inflexion and the size of potentiometric titration curves. As presented, the growing number of EO groups induces a more flattened titration curve and for this reason it is harder to find the end-point and it is more difficult to quantify the anionic surfactants in commercial samples.

#### 2.3.4. Titration of Technical Grade Surfactants

**Figure 6:**
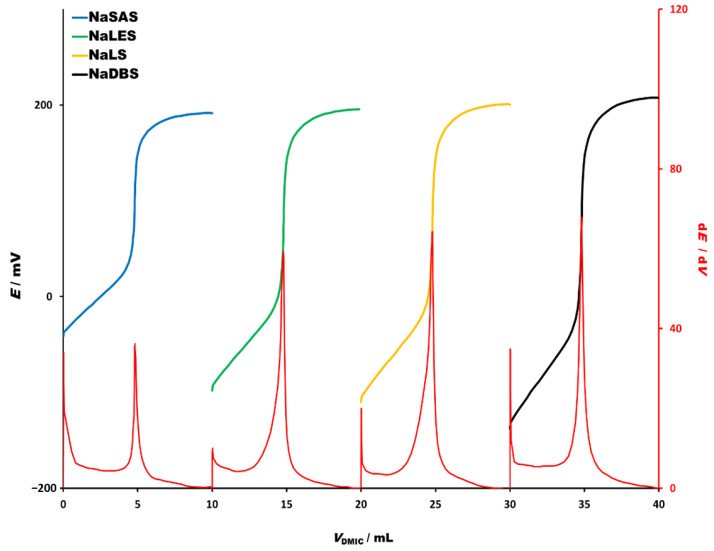
Potentiometric titration curves of technical grade anionic surfactants (4 × 10^−3^ M) with DMIC (4 × 10^−3^ M) as a titrant and the DHBI-TPB surfactant sensor as an end-point indicator. Corresponding first derivatives are presented in red lines below titration curves. The titration curves and their first derivatives are rearranged for the sake of clarity in the following order: SAS (blue line), LES (green line), SDS (yellow line), DBS (black line).

Potentiometric titrations of four technical grade anionic surfactants (4 × 10^−3^ M) with DMIC (4 × 10^−3^ M) as a titrant and the DHBI-TPB surfactant sensor as an end-point indicator are shown in Figure 6. The titration curves exhibit sharp inflexion points and high signal change with high first derivative signal change. Based on the titrations, the falling properties of selected technical grade anionic surfactants could be described as DBS > SDS > LES > SAS.

The accuracy of technical grade anionic surfactants determinations was estimated by the standard addition method, where an exact amount (30 µmol) of technical grade anionic surfactants was added to the sample titrated with DMIC (4 × 10^−3^ M) as a titrant and with the DHBI-TPB surfactant sensor as an end-point indicator (Table 3). As shown, experimentally found and added amounts for all four anionic surfactants, i.e., dodecyl sulfate, dodecyl benzenesulfonate, lauryl ether sulfate and secondary alkane sulfonate, were in good agreement and the recoveries varied from 99.5 to 101.3%. Proposed DHBI-TPB surfactant sensor was successfully employed for technical grade surfactant titrations.

#### 2.3.5. Titrations of Commercial Samples

Twelve commercial samples of detergents for household care, containing anionic surfactants, were used to quantify anionic surfactant content by DHBI-TPB surfactant sensor (Table 4). The developed DHBI-TPB surfactant sensor method was compared to ISE surfactant sensor and with a two-phase titration method. Three groups of detergents were analyzed: powdered (four samples), liquid-gel (four samples) and handwashing detergents (four samples). The pH was adjusted to 3 to avoid the interfering effect of amphoteric surfactants sometimes present in the product formulations.

The results for five independent repetitions were in good agreement and within expected errors for all the tested detergent formulations. Analyzed samples containing handwashing detergent formulations showed the highest anionic surfactant content from 13.98 to 15.89%, powdered detergent samples anionic surfactant content was from 5.75 to 6.88% and liquid gel detergents had 2.01 to 2.56% of anionic surfactants, respectively. Student’s *t*-test (at 95% confidence level) was applied to compare the difference between the results of the two-phase titration or ISE surfactant sensor and those obtained with the DHBI-TPB surfactant sensor. No significant difference was found between data sets.

## 3. Materials and Methods

### 3.1. Reagents and Materials

For direct potentiometric response measurements, anionic surfactant analytical grade dodecylsulfate (SDS) and technical grade dodecylbenzenesulfonate (DBS) (all from Fluka, Buchs, Switzerland), all sodium salts, were used. Analytical grade cationic surfactants used for titrations were benzethonium chloride (Hyamine 1622), cetylpyridinium chloride (CPC), hexadecyltrimethylammonium bromide (CTAB) and 1,3-didecyl-2-methylimidazolium chloride (DMIC) (all acquired from Merck, Munich, Germany).

Other technical grade anionic surfactants used for titrations were secondary alkane sulfonate (SAS, Hostapur SAS 60, Hoechst, Germany), dodecyl sulfate (Texapon LS 35, Cognis, Germany) and lauryl ether sulfate (LES, Texapon N 70, Cognis, Germany), all sodium salts.

Analytical grade alkane sulfonate homologues were sodium heptanesulfonate, sodium nonansulfonate and sodium undecanesulfonate (all acquired from Merck, Munich, Germany).

Nonionic surfactants were Genapol O 060, Genapol O 100 and Genapol O 200 (all from Clariant, Muttenz, Switzerland) with declared purity higher than 99%.

All salt solutions were prepared using analytical grade chemicals.

Twelve commercial detergents for household care (powdered, liquid-gel and handwashing) were purchased from the local stores.

### 3.2. Ionophore Characterization by FTIR

**Figure 7:**
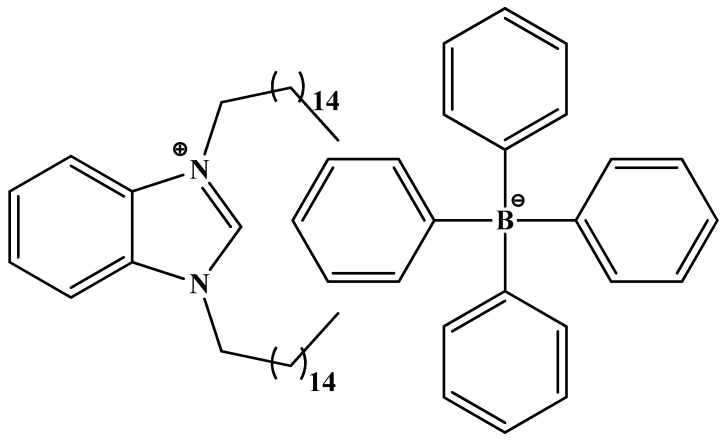
1,3-dihexadecyl-1*H*-benzo[*d*]imidazol-3-ium tetraphenylborate (DHBI–TPB ion-pair).

The DHBI–TPB ion-pair presented in Figure 7 was prepared by the procedure described earlier [16]. ATR-FT-IR spectrometer Spectrum Two (Perkin Elmer, Waltham, MA, USA) was used to characterize the DHBI–TPB ion-pair and to compare it with pure DHBI and TPB (Figure S1).

### 3.3. Computational Details of Ionophore Characterization

DHBI^+^ cation was parameterized through RESP charges at the HF/6–31G(d) level to be consistent with the employed GAFF force field, while TPB^−^ anion was considered according to literature recommendations [18]. The aforementioned ions were solvated in a 15 Å rectangular box of water, which allowed for 7.528 solvent molecules, calculated to match experimental solvent densities, and submitted to the geometry optimization in the AMBER 16 program [19] by employing periodic boundary conditions in all directions. Optimized systems were gradually heated from 0 to 300 K and equilibrated during 30 ps using NVT conditions, followed by productive and unconstrained MD simulations of 300 ns, employing a time step of 2 fs at a constant pressure (1 atm) and temperature (300 K), the latter held constant using a Langevin thermostat with a collision frequency of 1 ps^−1^. The nonbonded interactions were truncated at 11.0 Å. The binding free energies among components, Δ*G*_BIND_, were calculated using the established MM-PBSA protocol [19,20], all in line with our earlier reports on similar systems [21,22,23]. For that purpose, every second snapshot, 75.000 in total, collected from the entire MD trajectory, were utilized.

### 3.4. Preparation of Surfactant Sensor

The sensor membrane was prepared by the previously described procedure [16]. The high molecular weight PVC (33%) was mixed with a plasticizer (66%) and the DHBI-TPB ionophore (1%). After drying, the sensing membrane was inserted in the electrode body with 3 M NaCl inner electrolyte and used for further research.

### 3.5. Apparatus

Metrohm 794 Basic Titrino paired with Metrohm 781 pH/mV meter was used to measure response characteristics, pH influence and interferences. Metrohm 808 Titrando was employed for potentiometric titrations, nonionic surfactant influence study and real sample titrations. A Metrohm pH electrode was used to check the pH and a Metrohm silver/silver (I) chloride was used as a referent electrode for all investigations.

### 3.6. Procedure

#### 3.6.1. Potentiometric Sensor Characterization

To fully characterize the DHBI-TPB surfactant sensor on anionic surfactants, response measurements were performed. SDS and DBS anionic surfactants were incrementally added to deionized water and 0.01 M Na_2_SO_4_ solution (for high ionic strength study). Anionic surfactant concentrations of 4 × 10^−3^ M and 4 × 10^−4^ M were used to reach the logarithmic activity range from approximately −2 to −8.

Interference study was performed by incremental addition of SDS in 0.01 M interfering ion solution. A fixed interference method [17] to calculate the selectivity coefficient for all selected interfering anions was employed.

The pH influence on DHBI-TPB surfactant sensor properties was observed in the pH range 2–12. Corresponding amounts of 0.5 M HCl and 0.5 M NaOH were added to reach certain pH values.

After each measurement, surfactant sensor was washed with deionized water.

#### 3.6.2. Potentiometric Titrations

Potentiometric titrations were performed in dynamic equivalent point titration (DET) mode with signal drift 5 mV/min. Waiting time between increments was 15 to 30 s.

An aliquot of 5 mL anionic surfactant was added to 20 mL deionized water and used for titrations.

For titrations of SDS (4 × 10^−3^ M) four analytical grade cationic surfactants (4 × 10^−3^ M) were used: Hyamine 1622, CPC, CTAB and DMIC. Four analytical grade alkane sulfonate homologues (4 × 10^−3^ M) with different chain length—heptanesulfonate (7C), nonansulfonate (9C) and undecanesulfonate (11C), were used for titrations with DMIC (4 × 10^−3^ M).

The influence of nonionic surfactants on titrations of SDS (4 × 10^−3^ M) with DMIC (4 × 10^−3^ M) was investigated by the addition of nonionic surfactants with 6 EO, 10 EO and 20 EO groups and by varying 10 EO group nonionic surfactants in different molar ratios with SDS.

Four technical grade anionic surfactants (4 × 10^−3^ M)—SAS, LES, SDS and DBS, were used for titrations with DMIC (4 × 10^−3^ M). Twelve commercial detergents containing anionic surfactants were used for titrations with DMIC.

After each measurement, surfactant sensor was washed with deionized water.

## 4. Conclusions

The 1,3-dihexadecyl-1*H*-benzo[*d*]imidazol-3-ium-tetraphenylborate (DHBI-TPB)-based surfactant sensor was successfully applied for the quantification of anionic surfactants in commercial household detergent products. The DHBI-TPB surfactant sensor showed excellent resistance to interferences produced by the different organic and inorganic anions usually used in product formulations. The developed device could also be used in the broad pH range from pH 2–10. The DHBI-TPB ionophore was successfully characterized by ATR FT-IR. Computational analysis confirmed the formation of the DHBI-TPB ionophore in the aqueous solution with optimal exergonicity to allow for the beneficial analytical responses. In addition, it underlined C–H∙∙∙π interactions as crucial for the recognition of the components, surpassing electrostatic charge–charge attractions and π–π stacking interactions, both with moderate significance. DMIC was selected as the most reliable titrant for the use in potentiometric titrations with DHBI-TPB surfactant sensor as an end-point indicator. High solubility alkane sulfonate homologues with chain-lengths from 7 to 11 were successfully titrated with DMIC and DHBI-TPB surfactant sensor as an end-point indicator. Nonionic surfactants had a negative impact on titration curves, shape, inflexion and signal change of anionic surfactants when the EO concentration or the number of EO groups was increased. The titration curves appeared more flattened; in addition, the inflexion points were harder to detect from the first derivation curve. The DHBI-TPB sensor was successfully tested to measure technical grade anionic surfactants concentrations with recoveries from 99.5 to 101.3%. DHBI-TPB surfactant sensor was effectively employed for quantification of anionic surfactants in twelve samples of powered, liquid-gel and handwashing home care detergents. The results were compared with ISE surfactant sensor and a two-phase titration method and showed good agreement.

Thus, the DHBI-TPB surfactant sensor has advantages not only in terms of analytical properties, but also in terms of price, simplicity, speed and lack of need for the use of organic solvents or other additives, compared to the usually employed two-phase titration method. The DHBI-TPB surfactant sensor could be considered as a new and reliable analytical tool and has a promising perspective for its implementation in industry for quality control or in environmental monitoring.

## Figures and Tables

**Table 1:** Calculated response characteristics of DHBI-TPB surfactant sensor to anionic surfactants SDS and DBS measured in H_2_O and Na_2_SO_4_ aq., at wide concentration range, with mean values at ±95% confidence limits.

Parameters	Anionic Surfactant			
	SDS		DBS	
	In H_2_O	In SO_4_^2−^	In H_2_O	In SO_4_^2−^
Slope (mV/decade)	60.1 ± 0.5	59.7 ± 0.4	58.4 ± 0.6	58.7 ± 0.5
Correlation coefficient (R^2^)	0.9993	0.9994	0.9995	0.9995
Limit of detection (M)	3.2 × 10^−7^	4.2 × 10^−7^	6.1 × 10^−7^	6.1 × 10^−7^
Useful linear concentration range (M)	4.6 × 10^−7^ to 5.1 × 10^−3^	6.8 × 10^−7^ to 5.1 × 10^−3^	8.9 × 10^−7^ to 4.1 × 10^−3^	8.9 × 10^−7^ to 4.1 × 10^−3^

**Table 2:** Calculated logarithm of selectivity coefficient for different inorganic and organic anions (0.01 M) mostly used in product formulations, measured with the DHBI-TPB surfactant sensor for SDS.

Interfering Anions	$log\;K_{An_{i.}^{-}}^{pot}$
Chloride	−3.92
Carbonate	−4.03
Nitrate	−3.93
Acetate	−3.27
Sulfate	−4.69
Borate	−4.13
EDTA	−4.57
Dihydrogenphosphate	−3.75
Hydrogen carbonate	−3.30
Benzoate	−3.46
NaDBS	−0.10
Xylensulfonate	−3.48
Fluoride	−4.12
Bromide	−3.98
Hydrogen sulfate	−3.82

**Table 3:** Potentiometric titration results of some technical grade anionic surfactants with DMIC (4 × 10^−3^ M) as a titrant and with the DHBI-TPB surfactant sensor as an end-point indicator, with mean values at ±95% confidence limits.

Technical Grade Anionic Surfactant	*w* (Surfactant) */%	*n* (Added)/µmol	*n* (Found) **/µmol	Recovery/%	RSD/%
Dodecyl sulfate	92.51 ± 0.54	30	30.12 ± 0.07	100.4	0.22
Dodecyl benzenesulfonate	47.73 ± 0.21	30	30.22 ± 0.05	101.0	0.31
Lauryl ether sulfate	27.12 ± 0.09	30	29.85 ± 0.11	99.5	0.54
Secondary alkane sulfonate	67.41 ± 0.48	30	30.38 ± 0.11	101,3	0.76

* average on 5 determinations; ** average on 3 determinations.

**Table 4:** Results for potentiometric titration of commercial products containing anionic surfactants by the DHBI-TPB surfactant sensor compared with ISE surfactant sensor and a two-phase titration method.

Commercial Detergents		% Anionic Surfactant		
		DHBI-TPB	ISE Surfactant Sensor *	Two-Phase Titration **
Powdered	sample 1	6.14 ± 0.09	6.03	6.38
	sample 2	6.76 ± 0.15	6.88	6.86
	sample 3	5.78 ± 0.06	5.68	5.45
	sample 4	6.03 ± 0.07	6.11	6.08
Liquid-gel	sample 5	2.56 ± 0.07	2.49	2.66
	sample 6	2.33 ± 0.06	2.31	2.19
	sample 7	2.13 ± 0.11	2.09	2.01
	sample 8	2.01 ± 0.04	2.12	2.22
Handwashing	sample 9	15.89 ± 0.19	15.76	15.64
	sample 10	14.11 ± 0.11	14.14	14.31
	sample 11	13.98 ± 0.07	13.88	13.72
	sample 12	14.35 ± 0.21	14.41	14.48

* surfactant sensor presented in [15]; ** [5].

## Data Availability

The data presented in this study are available on request.

## Supplementary Materials

The following are available online, Figure S1: ATR-FT-IR spectra of cationic surfactant 1,3-dihexadecyl−1H-benzo[d]imidazol−3-ium bromide (DHBI-Br), sodium teraphenyl borate (Na-TPB) and ionophore 1,3-dihexadecyl−1H-benzo[d]imidazol−3-ium tetraphenylborate (DHBI–TPB), Figure S2: RMSD graphs during the molecular dynamics simulation of the DHBI+ cation (in black) and TPB– anion (in red) in the aqueous solution, revealing a much higher flexibility of the former, Figure S3: Time dependence of the distance between the boron atom in TPB– and the nitrogen atoms in DHBI+ during the molecular dynamics simulation, indicating complex formation in the equilibrium with dissociated components, Figure S4: RDF graph considering B(TPB–)∙∙∙N(DHBI+) distances during 300 ns of the molecular dynamics simulation 64with the maximum value located at around 5.4 Å, Figure S5: Charge distribution within the DHBI+ cation and the TPB– anion, either isolated or within the DHBI–TPB complex, as obtained through the NBO procedure at the (SMD)/M06–2X/6–31+G(d) level of theory. Particular set of atoms considered for the analysis is denoted with a color and includes the attached hydrogen atoms as well.

## Sample Availability

Samples of the compounds are available from the authors.
