# Supplementary material for: Potentiometric Surfactant Sensor Based on 1,3-Dihexadecyl-1H-benzo[d]imidazol-3-ium for Anionic Surfactants in Detergents and Household Care Products
Source: Molecules. 2021 Jun 14;26(12):3627. doi: 10.3390/molecules26123627 (PMC8231998; doi:10.3390/molecules26123627)
Supplement: Supplementary file 1 [file molecules-26-03627-s001.zip › molecules-1240136-supplementary.pdf]

molecules

MDPI

Article

Potentiometric Surfactant Sensor Based on 1,3-Dihexadecyl-1*H*-benzo[*d*]imidazol-3-ium for Anionic Surfactants in Detergents and Household Care Products

Nikola Sakač [1,*], Dubravka Madunić-Čačić [1,2], Dean Marković [3], Lucija Hok [4], Robert Vianello [4], Bojan Šarkanj [5], Bojan Đurin [6], Krunoslav Hajdek [7], Božo Smoljan [7], Stjepan Milardović [8], Brunislav Matasović [9], and Marija Joza-nović [9,*]

[1] University of Zagreb, Faculty of Geotechnical Engineering, 42000 Varaždin, Croatia; nikola.sakac@gfv.unizg.hr
[2] Saponia Chemical, Pharmaceutical and Foodstuff Industry, Inc., 31000 Osijek, Croatia; dubravka.madunic-cacic@saponia.hr
[3] University of Rijeka, Department of Biotechnology, 51000 Rijeka, Croatia; dean.markovic@biotech.uniri.hr
[4] Ruđer Bošković Institute, Division of Organic Chemistry and Biochemistry, 10000 Zagreb, Croatia; lucija.hok@irb.hr (L.H.); robert.vianello@irb.hr (R.V.)
[5] University North, Department of Food Technology, 48000 Koprivnica, Croatia; bsarkanj@unin.hr
[6] University North, Department of Civil Engineering, 42000 Varaždin, Croatia; bdjurin@unin.hr
[7] University North, Department of Packaging, Recycling and Environmental protection, 48000 Koprivnica, Croatia; krunoslav.hajdek@unin.hr (K.H.); bozo.smoljan@unin.hr (B.S.)
[8] University of Zagreb, Faculty of Chemical Engineering and Technology, 10000, Zagreb, Croatia; stjepan.milardovic@fkit.hr
[9] University of Osijek, Department of Chemistry, HR-31000 Osijek, Croatia; brunislav.matasovic@kemija.unios.hr (B.M.); mjozanovic@kemija.unios.hr (M.J.)
* Correspondence: nikola.sakac@gfv.unizg.hr; Tel.: +385915830336 (N.S.); mjozanovic@kemija.unios.hr; Tel.: +385996865716 (M.J.)

Supplementary material

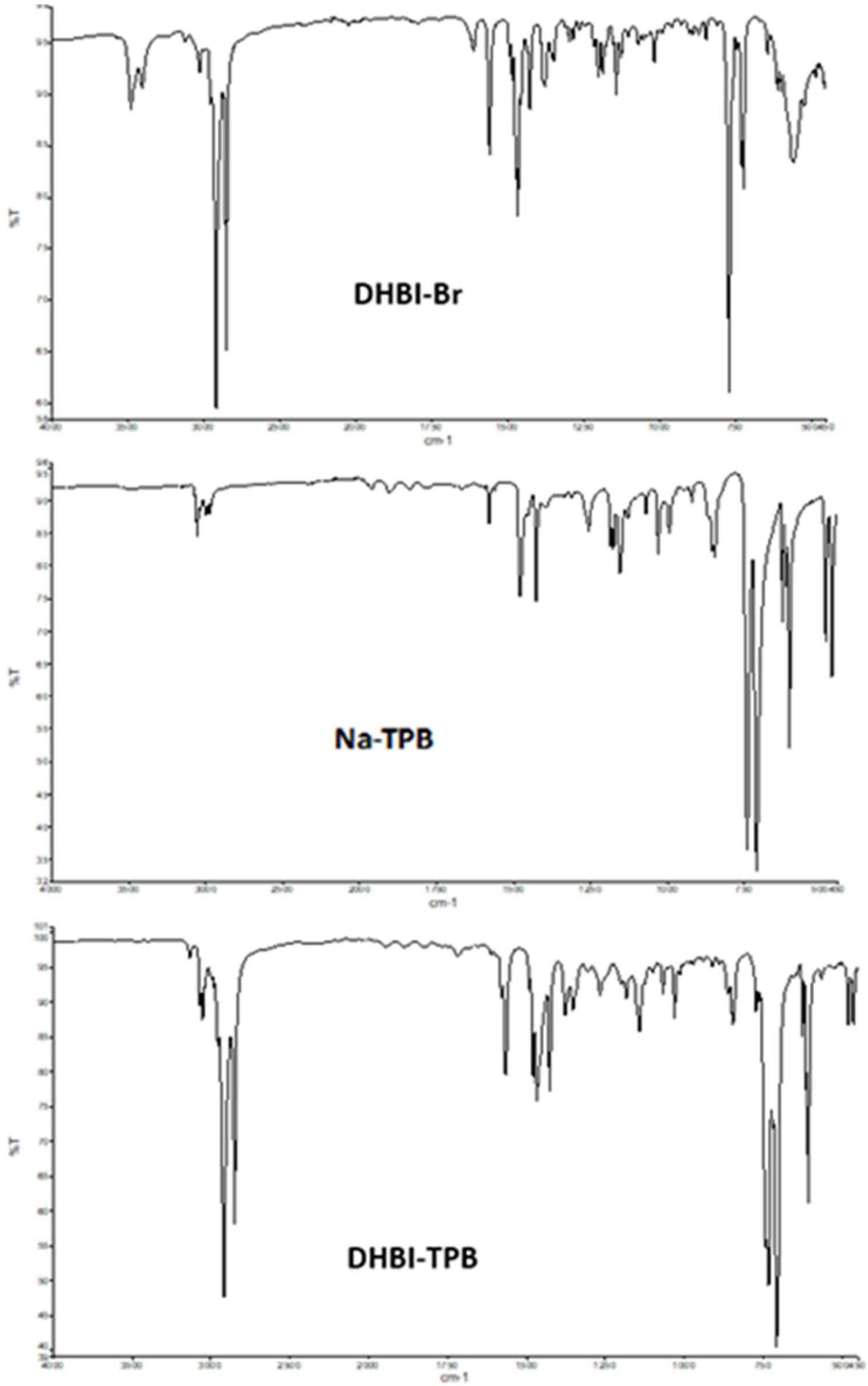

Figure S1. ATR-FT-IR spectra of cationic surfactant 1,3-dihexadecyl−1H-benzo[d]imidazol−3-ium bromide (DHBI-Br), sodium teraphenyl borate (Na-TPB) and ionophore 1,3-dihexadecyl−1H-benzo[d]imidazol−3-ium tetraphenylborate (DHBI–TPB).

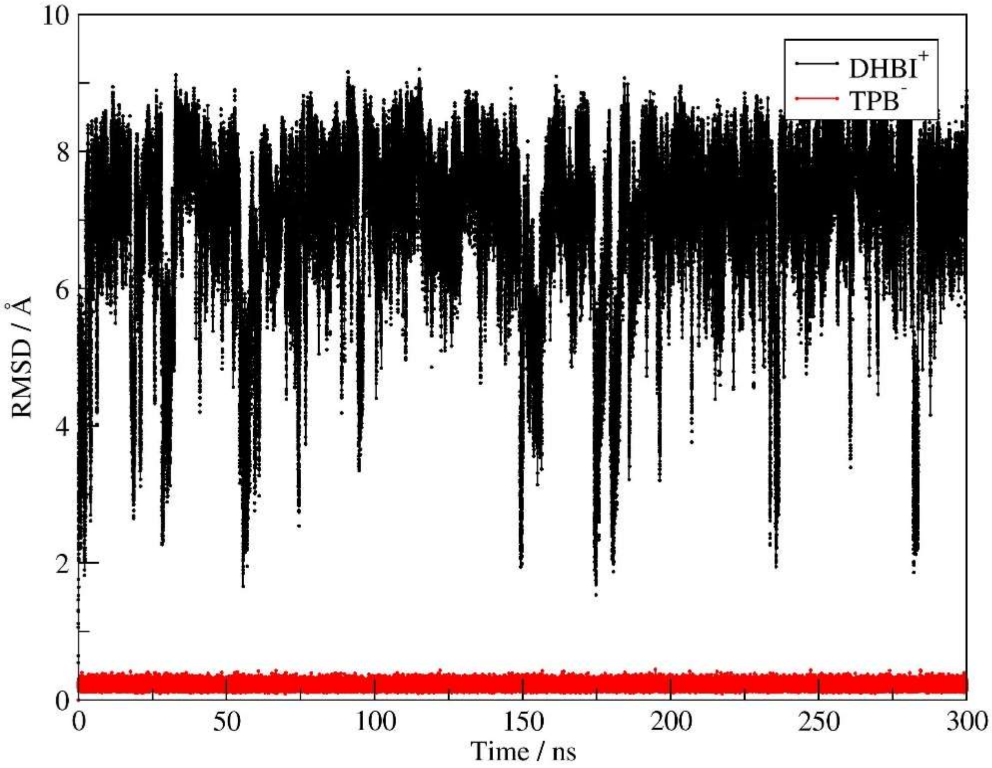

Figure S2. RMSD graphs during the molecular dynamics simulation of the $DHBI^+$ cation (in black) and TPB^- anion (in red) in the aqueous solution, revealing a much higher flexibility of the former.

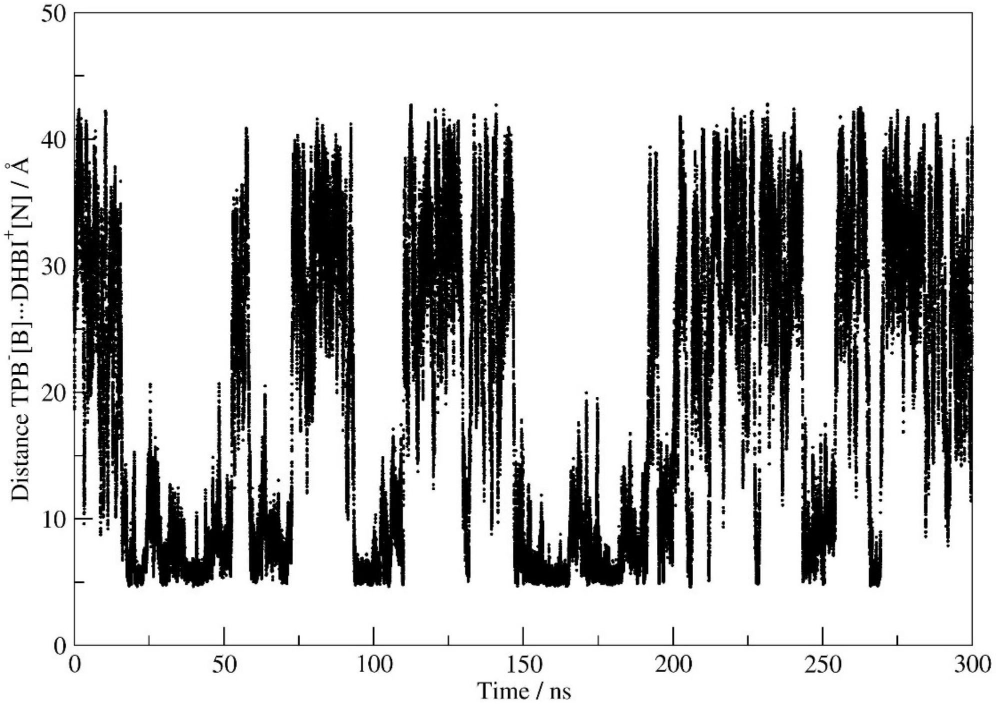

Figure S3. Time dependence of the distance between the boron atom in TPB^- and the nitrogen atoms in $DHBI^+$ during the molecular dynamics simulation, indicating complex formation in the equilibrium with dissociated components.

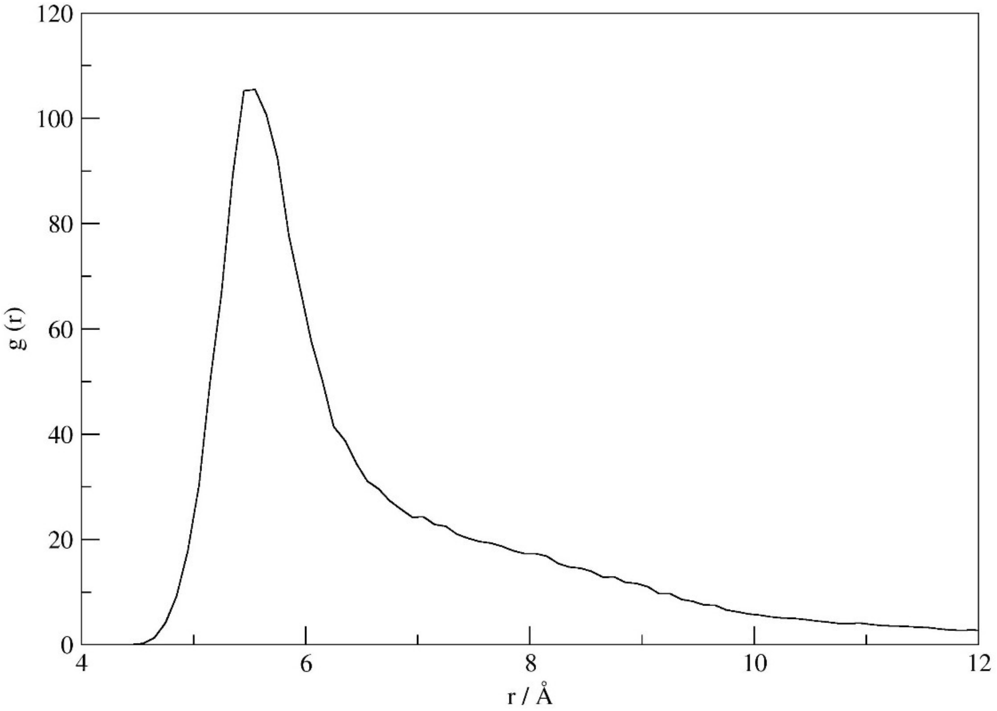

Figure S4. RDF graph considering B(TPB$^-$)···N(DHBI$^+$) distances during 300 ns of the molecular dynamics simulation with the maximum value located at around 5.4 Å.

ISOLATED COMPONENTS BEFORE THE COMPLEX FORMATION

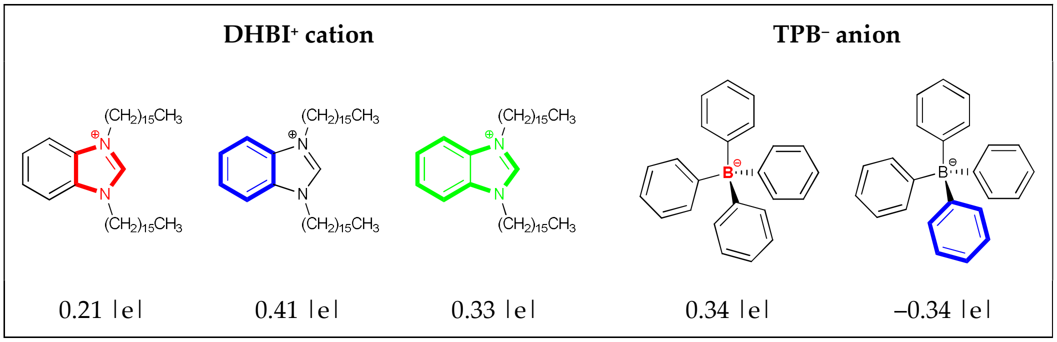

COMPONENTS WITHIN THE DHBI–TPB COMPLEX

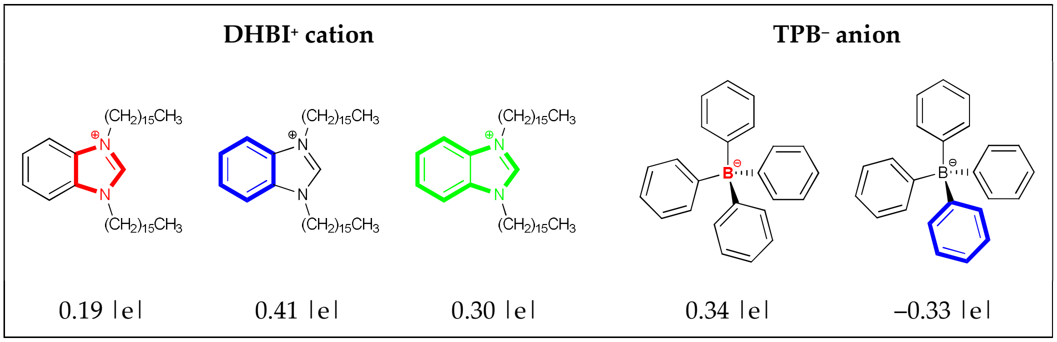

ENTIRE COMPONENTS WITHIN THE DHBI–TPB COMPLEX

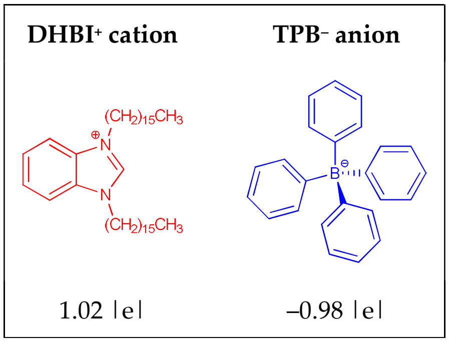

Figure S5. Charge distribution within the DHBI⁺ cation and the TPB⁻ anion, either isolated or within the DHBI–TPB complex, as obtained through the NBO procedure at the (SMD)/M06–2X/6–31+G(d) level of theory. Particular set of atoms considered for the analysis is denoted with a color and includes the attached hydrogen atoms as well.
